# eHealth tools to assess the neurological function for research, in absence of the neurologist – a systematic review, part I (software)

**DOI:** 10.1007/s00415-023-12012-6

**Published:** 2023-10-17

**Authors:** Vasco Ribeiro Ferreira, Esther Metting, Joshua Schauble, Hamed Seddighi, Lise Beumeler, Valentina Gallo

**Affiliations:** 1 Department of Sustainable Health, University of Groningen, Campus Fryslân, Wirdumerdijk 34, 8911 CE Leeuwarden, The Netherlands; 2 Faculty of Economics and Business, University of Groningen, Groningen, The Netherlands; 3 University Medical College Groningen, Groningen, The Netherlands; 4 Department of Knowledge Infrastructure, University of Groningen, Campus Fryslân, Leeuwarden, The Netherlands; 5 Department of Psychology, Faculty of Behavioural and Social Sciences, University of Groningen, Groningen, The Netherlands; 6 Department of Intensive Care, Medical Centre Leeuwarden, Leeuwarden, The Netherlands

**Keywords:** Epidemiology, Neurological Diseases, eHealth, Software, Neurological symptoms, Neurological signs

## Abstract

**Background:**

Neurological disorders remain a worldwide concern due to their increasing prevalence and mortality, combined with the lack of available treatment, in most cases. Exploring protective and risk factors associated with the development of neurological disorders will allow for improving prevention strategies. However, ascertaining neurological outcomes in population-based studies can be both complex and costly. The application of eHealth tools in research may contribute to lowering the costs and increase accessibility. The aim of this systematic review is to map existing eHealth tools assessing neurological signs and/or symptoms for epidemiological research.

**Methods:**

Four search engines (PubMed, Web of Science, Scopus & EBSCOHost) were used to retrieve articles on the development, validation, or implementation of eHealth tools to assess neurological signs and/or symptoms. The clinical and technical properties of the software tools were summarised. Due to high numbers, only software tools are presented here.

**Findings:**

A total of 42 tools were retrieved. These captured signs and/or symptoms belonging to four neurological domains: cognitive function, motor function, cranial nerves, and gait and coordination. An additional fifth category of composite tools was added. Most of the tools were available in English and were developed for smartphone device, with the remaining tools being available as web-based platforms. Less than half of the captured tools were fully validated, and only approximately half were still active at the time of data collection.

**Interpretation:**

The identified tools often presented limitations either due to language barriers or lack of proper validation. Maintenance and durability of most tools were low. The present mapping exercise offers a detailed guide for epidemiologists to identify the most appropriate eHealth tool for their research.

**Funding:**

The current study was funded by a PhD position at the University of Groningen. No additional funding was acquired.

## Background

Neurological disorders, including among others Alzheimer’s Disease and other dementias, Parkinson’s Disease, Multiple Sclerosis, epilepsy and headache, represent approximately 3% of the global burden of disease [1]. The burden of all neurological disorders combined has increased steadily since the early 1990s. The disability-adjusted life-years (DALYs) due to neurological conditions have increased by 15% worldwide in 2016 compared to 1990, despite the decline in communicable neurological disorders. Similarly, deaths by neurological disorders have increased by 39% in the same time period [2]. The highest incidence and mortality of neurological disorders are reported in low and middle-income countries, where they often coexist with limited clinical and research resources [2]. No curative treatment is currently available for the majority of neurological disorders, therefore prevention is essential to reduce the overall burden [3].

The use of electronic tools has become widely available at present, and information technology has played an increasingly prominent role in clinical medicine and research [4]. In this field, the various tools are collectively referred to as electronic health tools, or eHealth, in short. In general, eHealth tools contribute to improving assessment and intervention, closing the physical distance between patient and clinician, and assisting research [4, 5]. The use of eHealth tools may involve the presence of a skilled health worker (in-person or via video-conferencing) or be available as a fully automated tool or device, e.g. eHealth services that screen for disorders, as often seen for example in mental health [6]. eHealth tools can be divided into those that rely solely on software, and those using specific hardware. The tools in the first group (e.g., web-based, mobile app) have wider application as they rely solely on the availability of adequate support, i.e. a smartphone and/or a laptop. eHealth tools relying on specific equipment (i.e., a handle to measure grip strength), on the other hand, often require additional logistics, such as transportation and trained personnel. The development and use of eHealth tools became more relevant during the recent COVID-19 pandemic when access to in-person contacts was limited [7].

A large proportion of eHealth is used for diagnosis or disease management [8]. Nonetheless, some of these tools are extremely relevant for research, as well. In particular, eHealth tools collecting data outside hospital settings and without relying on specialised personnel are of particular interest for epidemiological studies [9]. Population-based epidemiological studies often require the assessment of clinical outcomes in large cohorts, and eHealth tools can enable data collection on a large scale. This is particularly relevant for studying hard-to-reach populations or large cohorts in low-income settings, where research-related resources can be scarce [10]. Among the eHealth tools available to be used for data collection, those focusing on the assessment of neurological function are particularly valuable. For research purposes only, a comprehensive eHealth assessment of the neurological function could potentially replace the assessment based on the neurological examination made by clinical neurologists, which is a very expensive resource. Capturing neurological signs and symptoms distribution at the population level might allow the estimate of the prevalence of selected neurological disorders in epidemiological studies.

Mapping and describing tools to be potentially used for research serves as a basis for the creation and implementation of novel eHealth tools in the field of neuroepidemiology. A comprehensive map, therefore, can be useful both for guiding epidemiological research and for the development of future tools. This systematic review aimed to capture and map eHealth tools capable of identifying any neurological sign and/or symptom in the general population (i.e., that can be used for epidemiological research, as opposed to their clinical application), currently available in the literature [11]. The intent was, therefore, to focus on the description and characterisation of these tools, rather than the studies in which they were used or the underlying populations. Given the large number of records found, only software tools were reported in this paper (i.e. eHealth tools that do not require extra equipment, other than a mobile device or computer), while hardware will be the focus of a future paper.

## Methods

A protocol for this systematic review was registered in the PROSPERO Database (ID: 314,489), and subsequently published [11].

### Search strategy and selection criteria

The search strategy was devised to capture all relevant papers. A total of four main fields were identified and linked with an AND Boolean connector: electronic tool (mobile app, electronic app, app, device, eHealth, mHealth, wearable), assessment (screening, assessment, measurement), sign and/or symptom (sign, symptom, outcome, disease, disorder), and neurological examination (neuro, brain, speech, tremor, cognitive, gait, motor, cranial, coordination, sensation). Within each field, similar terms were linked with an OR Boolean connector. An additional field containing terms capturing tools used for diagnostic or clinical purposes (i.e., intervention, improvement, rehabilitation, care, treatment) was defined and removed from the search by using a NOT Boolean connector. The search terms referring to the neurological symptoms/signs were based on a conventional neurological examination [12]. A full list of terms by field is reported in the protocol [11].

Searches were conducted on the 11th of February 2022, in four electronic databases: PubMed, Web of Science, EBSCOHost and Scopus. The searches were limited to the period from 2008 to date; 2008 was chosen as the year when the first modern smartphone was released, to capture only tools in line with contemporary technology.

The inclusion and exclusion criteria were defined according to an adapted version of the Population Intervention Control Outcome (PICO) criteria.

*Population* – Studies with human participants of every age, sex and gender were included. *Intervention* – tools that could be used outside clinical settings and without the assistance of a clinical neurologist in the process of data collection (i.e. tools to be used in research and not in clinical practice).

*Outcome* – Studies addressing the development, validation, or implementation of software eHealth interventions that assess a neurological sign, symptom or function.

Only empirical research published in English in peer-reviewed journals was considered. Animal studies, and studies using Artificial Intelligence or automated analysis to make a diagnosis were excluded. Likewise, studies that collected data using non-portable equipment (e.g., neuroimaging), lab procedures (e.g., biomarkers), or specialised medical personnel were excluded to identify solely the tools for epidemiological research and not clinical practice. When more than one paper reported data on the same tool, only the paper reporting data coming from the largest population was summarised in tables.

Further detail on eligibility can be found in the protocol [11].

### Data analysis

The Zotero software was used to store references and relevant information on each publication. Reference lists obtained from each search engine were combined, and duplicates removed. For initial eligibility purposes, titles and abstracts were screened. Subsequently, two reviewers independently assessed the inclusion/exclusion criteria of identified papers. Whenever there was a disagreement on the inclusion or exclusion of a given paper, a third reviewer offered their input, solving the disagreement.

Data extraction was structured according to the following categories:

- General characteristics of the paper: authors, year of publication, country;
- Type of study: development, validation, or implementation of electronic tools;
- eHealth tool: name, length of assessment, internet connection requirement, self-assessment vs. instructor-mediated assessment, validated vs. non-validated in a population, availability (i.e., platform);
- Participants: sample size, mean age and gender distribution if applicable;
- Context: setting of the research, source of funding;
- Outcome: sign/symptom assessed, type of output variable (e.g., score, measurement on a continuous scale);
- Technical characteristics and availability: licensing status, maintenance strategy, accessing link.

Corresponding authors were contacted to complement data provided by the published paper, where needed.

Included papers were not formally assessed in terms of their quality given the very high heterogeneity of the published article for their reporting. However, the quality of the descriptive papers and their validation studies, such as for example validation measures and group comparisons, were taken into consideration when summarising the results. eHealth tools were considered still active if a URL or another access mode was found to access them. All sections of the systematic review were reported following the Preferred Reporting Items for Systematic Reviews and Meta-Analyses (PRISMA) guidelines [13].

The information extracted from the original papers was reported in a series of tables aimed at providing an overview of relevant items at a glance, by technical characteristics, and by sign/symptom assessed. In addition, a conceptual graph mapping each tool by neurological function assessed was drawn using Visio Microsoft Software [14].

### Role of the funding source

The funder of the study had no role in study design, data collection, data analysis, data interpretation, or writing of the report.

## Findings

**Fig. 1:**
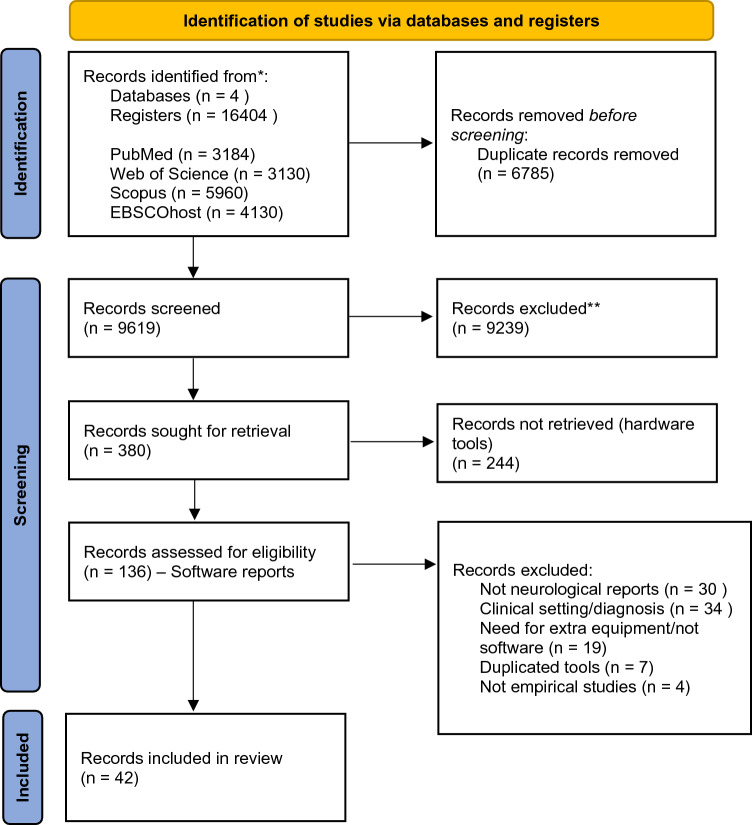
PRISMA flow diagram showing the process leading to the current sample of included papers

A total of 16,404 papers were initially obtained from the database searches. After duplicate removal, 9,619 papers remained to be screened. After excluding non-relevant items through titles and abstracts, a total of 380 reports were considered for inclusion. Of these, full texts were retrieved for the 136 papers reporting on software tools. After applying the inclusion and exclusion criteria, a total of 94 papers were excluded, leading to a final sample of 42 software eHealth tools included in the present review (Fig. 1). Reasons for exclusion were a) did not refer to a neurological sign or symptom (*n* = 30), b) did not refer to tools suitable for a research setting (*n* = 34), c) did not refer to a software tool, or required extra equipment (*n* = 19), d) duplicated tools (*n* = 6) or e) non-empirical studies (*n* = 4).

**Fig. 2:**
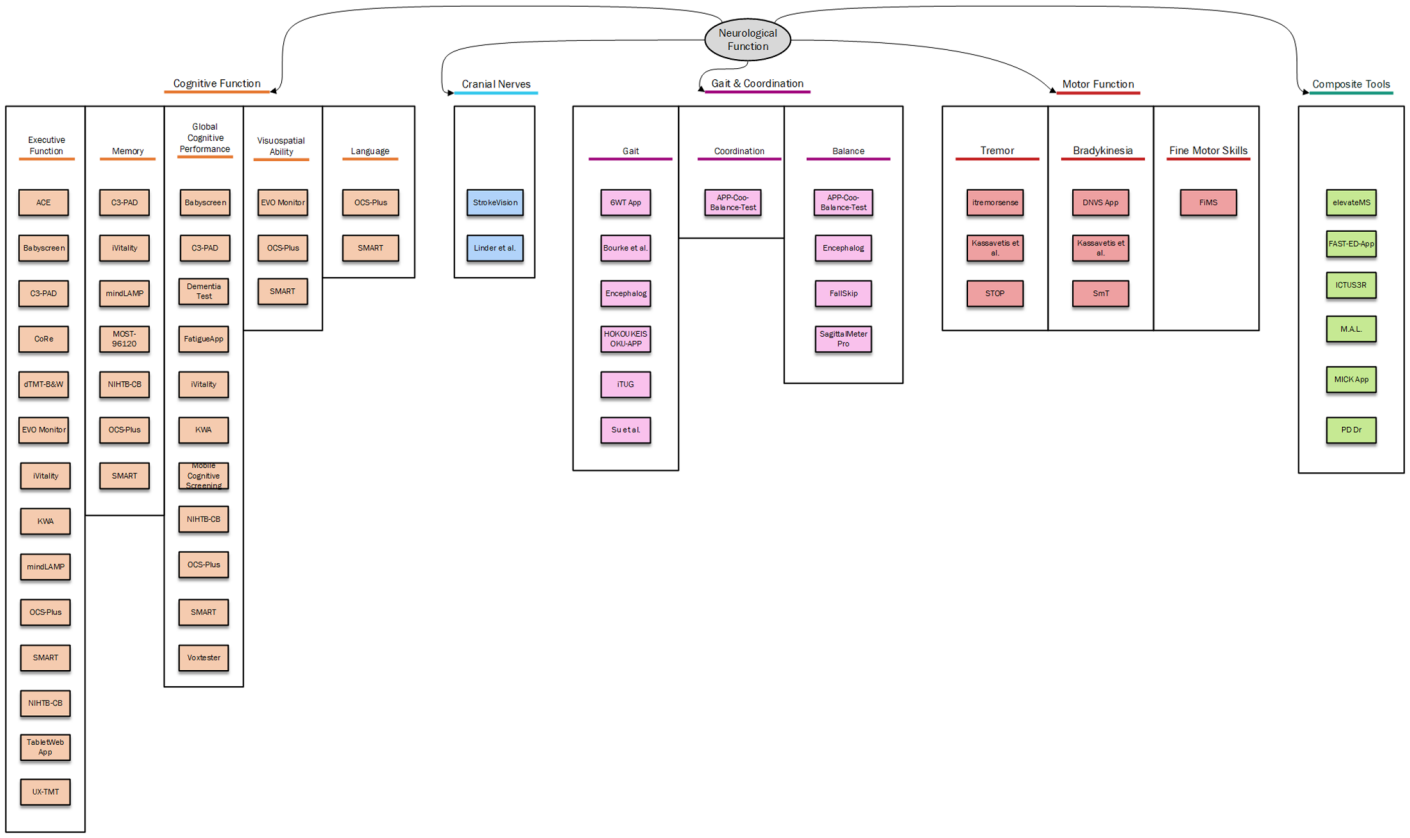
Mapping exercise of the captured tools, organised by neurological function and symptom and sign assessed

The main clinical characteristics of the tools are reported in Table 1, according to the neurological function assessed: 19 tools assessing the cognitive function [15–33]; six tools assessing the motor function [34–39]; two tools assessing cranial nerve function [42, 43]; and nine tools assessing gait and coordination [44–52]. In this table, the tools are organised by symptom or sign assessed (i.e. hand tremor), and the type of measurement used for assessment (i.e. measure of tremor intensity). In addition, general information on their validation is reported. There is an additional section in which a total of six composite tools [53–58], i.e. tools screening for a wider set of signs and/or symptoms in patients with a specific neurological condition (e.g., elevateMS in Multiple Sclerosis [53]) were reported. The technical properties of all the tools were summarised in Table 2, where information such as the need for an internet connection, or in which platform (Android OS or iOS) the tool is available from, were collated. A conceptual map displaying all the captured eHealth tools organised by neurological function is shown in Fig. 2.

**Table 1:** Clinical properties and measures of the captured eHealth tools to assess neurological function

Tool Name	Components screened	Validation and measures	Sample Age Mean (SD) or Median (IQR)/Range	Sample size (Comparison)	Measurement Unit/Output Variable
Cognitive function tools					
Adaptive cognitive evaluation (ACE) [15]	CI in MS: Attention and Processing Speed	Partially validated. Healthy vs. MS patients with and without cognitive impairment, against standard measures (SDMT, PASAT)	HC: 46.04 (3.72); MS CI: 50.87 (2.51); MS no CI: 52.68 (2.35)	77 participants (3 groups)	Numerical mean scores for attention, and RT in milliseconds
Babyscreen [16]	CI in children: Precision and Processing Speed	Partially validated. Healthy children, performance differentiated between age group according to scores obtained	Median (IQR)–31 months (26–34)	112 participants (2 groups)	Total item completion, speed and accuracy
C3-PAD [17]	CI: EM, WM and Processing Speed	Validated. Healthy participants vs. standardized neuropsychological tests: ANART, TMT, VFDT, ADCS PACC, FCSRT, MMSE, and SDMT	71.2 (7.6)	49 participants (1 group)	Composite Z-scores for each subtest
Cognition reaction (CoRe) [18]	CI in MS: Precision and Processing Speed	Validated. Healthy vs. MS patients, against paper-and-pencil version of the SDMT	HC: 38.1 (11.9); MS: 44.0 (11.0)	147 participants (2 groups)	Numerical scores for accuracy, and RT in milliseconds
DementiaTest [19]	CI in Dementia: Orientation	Not validated. Proposed development only. Based on the 6-CIT and SCID	NA	NA	Numerical scores with predefined cut-offs
Digital TMT Black and White (dTMT-B&W) [20]	CI: EF	Partially validated. Healthy vs cognitively impaired, against paper-and-pencil versions showing concurrent validity	HC: 53.0 (1.5); MCI – 67.2 (6.0)	44 participants (2 groups)	Scores for typical TMT (errors, total time)
EVO Monitor [21]	CI in MS: Visuomotor Tracking, Perceptual Discrimination, Multitasking	Partially validated. Healthy vs. MS patients with and without cognitive impairment, against standard measures: BICAMS – SDMT and MS Functional Composite + MRI	HC: 46.0 (3.7); MS CI: 49.0 (2.3); MS no CI: 53.8 (1.4)	124 participants (3 groups)	Numerical scores for each task
FatigueApp [22]	Fatigue in MS: EF, Memory and Verbal Fluency	Not validated. MS patients, against PROMIS short form	49.0 (10.9)	32 participants (1 group)	Numerical scores following PROMIS
iVitality [23]	CI in Dementia: EF and Memory	Partially validated. Healthy but at risk of Dementia participants, against standard measures (MMSE, 15-WVLT, TMT A&B and Stroop-Color-Word)	57.3 (5.3)	151 participants (1 group)	Numerical scores for each task, errors and total time for TMT
Karolinska WakeApp (KWA) [24]	CI in Sleep Deprivation: EF, EM and WM	Not validated. Healthy participants were assigned into groups (sleep deprived, non-deprived) and completed the test 3 times	Non-deprived: 25.3 (6.8); Deprived: 25.4 (5.2)	182 participants (2 groups)	Numerical scores for each task
MindLAMP [25]	CI in PD: EF	Validated. Healthy participants, against UPDRS, MoCA, Trails-B and WMS-III spatial span	63.2 (8.7)	27 participants (1 group)	Numerical scores, errors and total time for Trails
Mobile Cognitive Screening [26]	General Cognitive Screening in Dementia	Validated. Healthy vs. dementia patients, against paper-and-pencil measures (MoCA, MMSE) and computerised measures (ANAM, CANS-MCI, CANTAB, CNVS, CNTB, CGS, CSI, MCIS, MicroCog & Mindstreams)	HC: 72.6 (9.6) Dementia: 81.8 (4.8)	23 participants (2 groups)	Numerical general score out of 33
MOST-96120 [27]	CI: Memory and Orientation	Not validated. Patients with diverse diagnosis, against paper-and-pencil MOST, WAIS, Logical Memory-II & Visual Reproduction-II (WMS), delayed recall of 12-item Shopping List Test, 8-item proverb interpretation test (D-KEFS), and MMSE	76.8 (7.4)	98 participants (1 group)	Mean score for the memory orientation test
NIHTB-CB [28]	CI: EF and WM	Not validated. Healthy veterans (web-first vs iPad first), against web-based version of the battery – Dimensional Change Card Sorting Test, Flanker Inhibitory Control and Attention Test, List Sort Working Memory Test, Pattern Comparison Processing Speed	Web first: 41.6 (9.0); iPad first: 39.0 (9.2)	49 participants (2 groups)	Numerical scores for each task
Oxford cognitive screen-plus (OCS-Plus) [29]	General Cognitive Screening	Validated. Healthy participants, against ACE-R, CERAD, ROCF and Star Cancellation Test	62.7 (13.8)	320 participants (1 group)	Numerical scores for each task
SMART [30]	General Cognitive Screening and Language	Validated. Healthy vs MCI patients, against neuropsychological tests – Number Span Forwards, TMT-A, Stroop, Immediate/Delayed Recall, Benson Complex Figure Delayed Recall, Multilingual Naming Test, Category/Phonemic Fluency, and MoCA	HC: 72.4 (5.2); MCI: 74.0 (6.0)	69 participants (2 groups)	Composite Z-scores for each function
TabletWebApp [31]	CI in Dementia/MCI: EF, Visual Perception	Validated. Healthy vs MCI vs Dementia patients, against paper-and-pencil versions of TMT-A & B, and Bells Test	HC: 76.2 (4.2); MCI: 78.0 (5.4); Dementia: 78.6 (4.0)	83 participants (3 groups)	Numerical scores, errors and total time for TMT
UX-TMT [32]	CI in Dementia/PD: EF	Validated. Healthy vs PD patients vs MCI/Dementia patients, against MMSE-J, MoCA-J, SF-12 & PANAS20-J	HC: 55.8 (13.7); PD: 68.6 (6.7); MCI/D: 79.4 (7.6)	84 participants (3 groups)	Errors and total completion time
Voxtester [33]	Language/Speech impairment in PD	Not validated. Healthy young vs healthy elderly vs PD patients, against UPDRS speech scores	Median (IQR) – HYoung: 20.8 (19–29); HElder: 67.1 (60–77); PD: 67.2 (40–80)	65 participants (3 groups)	Numerical scores for error rate in word reading; speech duration time, number of words read, and characters per second
Motor function tools					
DNVS App [36]	Upper Extremity Bradykinesia and Processing Speed	Partially validated. PD patients, against vMDS-UPDRS III	68.4 (7.8)	23 participants (1 group)	Numerical scores for the two tasks
FiMS [37]	Fine Motor Skills: Tapping a moving target, dragging a target to a goal, moving a target through a maze, drag two separate targets	Validated. Healthy vs Myelopathic patients, against the mJOA	HC: 58.0 (9.1) Myelopathic: 60.5 (9.3)	93 participants (2 groups)	Numerical scores for each of the four tasks
Itremorsense [38]	Upper limb tremor in PD	Validated. Healthy vs PD patients (+ PD hospitalised), against gold standard UPDRS	HC: 67.2 (6.3); PD: 70.9 (11.8); PH hospitalised: 80.0 (4.2)	45 participants (3 groups)	Quantification of tremor using a sensor
Kassavetis et al. [39]	Motor Symptoms in PD (tremor, bradykinesia)	Partially validated. PD patients, against MDS-UPDRS	Median (IQ R) – 54.7 (34–75)	14 participants (1 group)	Numerical scores for the two screened components
Smartphone Tapper (SmT) [40]	Bradykinesia in PD	Validated. Healthy vs PD patients, against gold standard UPDRS + two mechanical tappers	HC: 53.4 (14.8); PD: 65.4 (9.0)	144 participants (2 groups)	Kinematic measurements: total distance finger movement, inter-tap distance, inter-tap dwelling
Sentient Tracking of Parkinson’s (STOP) [41]	Hand Tremor Intensity	Partially validated. PD patients, against UPDRS-II general score and Tremor item on UPDRS	Range – 52–73	11 participants (1 group)	Measures for tremor intensity
Visual acuity tools					
Linder et al. [42]	Visual Impairment in mTBI	Validated. Healthy participants, against clinical/ruler measurements (reading point of convergence, reading fluency and comprehension)	21.9 (2.0)	50 participants (1 group)	Numerical scores for static and dynamic visual acuity, and other mentioned tasks
StrokeVision [43]	Visual Acuity in Stroke	Validated. Stroke patients, against the gold standard for formal kinetic perimetry (GVFT or OVFA) + pencil-and-paper tests of inattention (Albert’s Test, Star Cancelation Test and Line Bisection)	Median (IQR) – 63 (54–72)	48 participants (1 group)	Screenshot to be assessed by a clinician
Gait and coordination tools					
6WT app [44]	Spine Abnormality: Back and lower-extremity pain, walking abnormality	Validated. Healthy participants, against PROMs – VAS for low-back and lower-extremity pain, COMI, ZCQ, and subjective walking distance and duration	44.2 (18.6)	330 participants (1 group)	Numerical overall score for 6WT test, measures for walking distance and duration
APP-Coo-Balance-Test [45]	Balance and Coordination impairment: static and dynamic balance	Partially validated. Healthy vs Cerebellar Ataxia patients, against BBS and SARA + validated static balance evaluating systems	HC: 39.3 (11.4); Cereb. Ataxia: 40.7 (10.9)	120 participants (2 groups)	Quantification of Static and Dynamic balance with sensor
Encephalog [46]	Fall Occurrence, stand-up time and mediolateral sway	Not validated. PD patients (fallers + non-fallers), against UPDRS-III scoring, Hoehn and Yahr Scale, and disease duration	Median (IQR) – Non-fallers: 66 (58.5–71.8); Fallers: 53.5 (50.5–60.3)	33 participants (2 groups)	Scores for the timed-up-and-go test (stand-up time, mediolateral sway), and fall occurrence
Bourke et al. [47]	Gait impairment: spatial and spatiotemporal characteristics	Not validated. Healthy vs MS patients, gait differentiating measures	HC: 34.9 (9.3): MS: 39.5 (7.9)	101 participants (2 groups)	Smartphone sensor measures for spatial and spatiotemporal gait characteristics
FallSkip [48]	Balance, Gait & functional abnormalities in PD	Not validated. Healthy vs Mild to Moderate PD patients, against no gold standard	HC: 67.2 (8.2); PD: 68.9 (9.0)	60 participants (2 groups)	Smartphone sensor data quantification
Hokoukeisoku-app [49]	Gait impairment: speed and daily cycle	Not validated. Does not screen for impairment (yet), simply quantifies gait	48.8 (12.8)	186 participants (1 group)	Scores for Daily Gait Speed, Daily Gait Cycle, Average of Daily Cadence
iTUG [50]	Gait impairment: standing, walking, turning, sitting	Not validated. Healthy vs Probable idiopathic normal-pressure hydrocephalus, against manual Timed-up and Go	HC: 79.4 (7.0); Idiopathic: 77.6 (5.5)	119 participants (2 groups)	Total time for task; stand, go, turn, come, and sit times
Sagittalmeter pro [51]	Balance Impairment: lumbar lordosis, pelvic tilt, sacral slope and pelvic incidence	Validated. Spine radiography patients, against radiograph examination by 3 experts and the Web-based PACS	50.9 (17.6)	30 participants (1 group)	Scores for several measurements – Lumbar lordosis, Pelvic Tilt, Sacral slope, and Pelvic incidence
Su et al. [52]	Gait impairment in PD: walking normally (single task) and while performing a serial-subtraction (dual task)	Validated. PD patients, against walking assessment, UPDRS-III, and MoCA	63.0 (10.0)	52 participants (1 group)	Scores for gait (single task), and while performing a serial-subtraction (dual task)
Composite tools					
ElevateMS [53]	MS Signs and Symptoms: finger tapping, walking and balance, and cognition	Not validated. Healthy vs MS patients (self-reported + clinically referred), against baseline assessments – PDDS, Cognitive, Upper & Lower Extremity assessment + functional tests (finger-to-nose, walk and balance)	HC: 39.3 (11.4); Self-reported: 45.2 (11.6); Clinically referred: 48.9 (11.2)	629 participants (3 groups)	Numerical scores for performance in each task: finger tapping, finger to nose, walk and balance, Digit Symbol Substitution Test
Fast-ED App [54]	Large Vessel Occlusion: Stroke Signs and Symptoms	Validated. Stroke patients, against FAST-ED original, RACE, CPSS, 3-ISS, and NIHSS	NA	2815 participants (retrospective data)	Numerical overall score
ICTUS3R [55]	Stroke signs: facial palsy, asymmetric arm weakness, speech and visual disturbance, headaches	Not validated. Tool based on the CPSS	NA	NA	Numerical overall score estimating the absolute risk of stroke
M.A.L [56]	PD Signs and Symptoms: speech, posture, gait, finger tapping, and processing speed	Validated. Healthy vs PD patients, against gold standard (UPDRS)	HC: 57.7 (14.3); PD: 65.1 (9.8)	20 participants (2 groups)	Scores for each of the five tasks: Voice (voice measures), Posture, Gait (acceleration, movement measures), Finger tapping (position and RT), Reaction Time in milliseconds
MICK App [57]	Concussion Signs and Symptoms: Naming Pictures and Numbers	Not validated. Healthy participants, against paper-and-pencil instrument counterparts – MULES and SUN	39.0 (16.0)	59 participants (1 group)	Numerical scores for the two tasks
PD Dr [58]	Hand tremor, walking and turning	Partially validated. PD patients, previously screened with UPDRS	68.5 (9.5)	40 participants (1 group)	Uses motion data from smartphone 3D accelerometer to differentiate performance (hand tremor test, walking test, turning test)

*6CIT* six-item cognitive impairment test, *ACE-R* Addenbrooke’s cognitive examination revised, *ADCS* PACC alzheimer’s disease cooperative study preclinical alzheimer cognitive composite’, *ANAM* automated neuropsychological assessment metrics, *ANART* American national adult reading test, *ICAMS* brief international cognitive assessment for MS, *CANS-MCI* computer-administered neuropsychological screen for mild cognitive impairment, *CANTAB* cambridge neuropsychological test automated battery, *Cerad* consortium to establish a registry for Alzheimer’s disease, *CGS* CogState computerised cognitive test battery, *CNTB* computerised neuropsychological test battery, *CNVS* computerised neurocognitive vital signs, *CSI* cognitive stability index, *D-KEFS* delis-kaplan executive function system, *EF* executive function; EM episodic memory; *FCSRT* free and cued selective reminding test, *MCI* mild cognitive impairment, *MCIS* mild cognitive impairment screen, *MMSE* mini mental state examination, *MoCA* montreal cognitive assessment, *MOST* memory orientation screening test, *MRI* magnetic resonance imaging, *MS* multiple sclerosis, *PASAT* paced auditory serial addition test, *PD* Parkinson’s disease, *PROMIS* patient-reported outcomes measurement information system, *ROCF* rey-osterrieth complex figure test, *RT* reaction time, *SCID* structured clinical interview for DSM disorders, *SDMT* symbol digit modalities test, *SF-12* 12-item short form survey, TMT trail making test, *UPDRS* unified parkinson’s disease rating scale, *VFDT* visual form discrimination Test, *WM* working memory, *WMS* wechsler memory scale, *WVLT *word verbal learning test, *HC* healthy controls *CI* cognitive impairment, *MS* multiple sclerosis, *mJOA* modified Japanese orthopaedic association scale, *PD* parkinson’s disease, *(v)MDS-UPDRS III* (virtual) movement disorder society-unified parkinson’s disease rating scale III, *GVFT *goldmann visual field test, *mTBI *mild traumatic brain injury, *OVFA* octopus visual field assessment, *BBS* berg balance scale, *COMI* core outcome measures index, *MoCA* montreal cognitive assessment, *PACS* picture archiving and communication system, *PD* Parkinson’s disease, *PROMs* patient reported outcome measures, *SARA* scale for the assessment and rating of ataxia, *UPDRS* unified Parkinson’s disease rating scale, *VAS* visual analog scale, *ZCQ* zurich claudication questionnaire, *3-ISS* 3-item stroke scale, *CPSS* cincinnati prehospital stroke scale, *FAST-ED* field assessment stroke triage for emergency Destination, *MS* multiple sclerosis, *MULES* mobile universal lexicon evaluation system, *NIHSS* national institutes of health stroke scale, *PD* parkinson’s disease, *PDDS* patient determined disease steps, *RACE* rapid arterial occlusion evaluation, *SUN* staggered uneven number naming, *UPDRS* unified parkinson’s disease rating scale

**Table 2:** Technical properties of the captured tools, organised by neurological function

Tool name	Internet requirement	Self-assessment	Length of assessment	Platform used	Country of study (language)
Cognitive function tools					
Adaptive cognitive evaluation (ACE)	Yes	Yes	Approximately 30 min	Platform not specified; Tablet-based	United States of America (English)
Babyscreen	Unknown	Instructor aided	Unknown	Platform not specified, administered on iPad	Ireland (English)
C3-PAD	No	Instructor explains, self-assessment after	Approximately 30 min	Developed for iOS 11, administered on iPad	United States of America (English)
Cognition reaction (CORE)	No	Instructor present in a study but not needed for the platform itself	Approximately 90 s	Platform not specified, administered on iPad	United Kingdom (English)
Dementiatest	Yes	Yes, or caregiver/family member	Unknown	Android OS and iOS	New Zealand (English)
Digital TMT Black and White (dTMT-B&W)	Unknown	Yes	Approximately 5 min	Android OS	South Korea (English and Korean)
EVO Monitor	Unknown	Yes	Approximately 7 min	Platform not specified; administered on iPad	United States of America (English)
FatigueApp	Yes	Instructor explains, self-assessment after	15 to 20 min	Web-based	United States of America (English)
iVitality	Yes	Yes	Unknown	Web-based, available for smartphone	The Netherlands
Karolinska WakeApp (KWA)	Unknown	Instructor practices with the participant first. Self-assessment after	Approximately 10 min	Web-based, but runs safely on Safari for iPhone and Chrome for Android	Sweden and Denmark
mindLAMP	Unknown	Instructor explains first, then it's self-assessment	Approximately 10 min	Android OS and iOS	United States of America (English)
Mobile cognitive screening	Unknown	Yes	Unknown	Android OS	Turkey (Turkish)
MOST-96120	Yes	Instructor aided	Approximately 30 min	iOS	United States of America (English)
NIHTB-CB	Unknown	Instructor aided	Unknown	Platform not specified, administered on iPad	United States of America (English)
Oxford cognitive screen-plus (OCS-Plus)	No	Instructor aided	Approximately 25 min	Android OS	Germany (German, Shangaan and English)
SMART	Yes	Yes	Approximately 5 min	Web-based	United States of America (English)
TabletWebApp	Yes	Yes, with vocal automatised instructions	Approximately 15 min	Web-based	Italy (Italian, English)
UX-TMT	Unknown	Yes, but the instructor is present for clarifications	Approximately 10 min	Android OS	Japan (Japanese)
Voxtester	Yes	Instructor aided	Unknown	Web-based, available for Android	Italy (Italian)
Visual acuity tools					
Linder et al	Unknown	Instructor aided	Unknown	Developed for iOS 11, administered on iPad	United States of America (English)
StrokeVision	Unknown	Unknown	Approximately 7 min	Android OS	United Kingdom (English)
Gait and coordination tools					
6WT app	Unknown	Yes	Approximately 8 min	Android OS and iOS	Switzerland (English, French and German)
APP-Coo-Balance-Test	Unknown	Instructor aided	Approximately 10 min	Android OS and iOS	Italy
Bourke et al	Unknown	Yes	Approximately 2 min	Android OS	Switzerland
Encephalog	Unknown	Yes	Approximately 5 min	Android OS and iOS	Italy
FallSkip	Unknown	Instructor aided	Approximately 15 min	Android OS	Spain
Hokoukeisoku-APP	Unknown	Yes	Unknown	Android OS and iOS	Japan
iTUG	Yes	Instructor aided	Approximately 7 min	iOS	Japan
SagittalMeter Pro	Unknown	Instructor aided	Approximately 1 min	Android OS and iOS	South Korea
Su et al	Yes	Yes	Unknown	iOS	China
Motor function tools					
DNVS App	Unknown	Yes	Unknown	iOS	United States of America (English)
FiMS	Unknown	Instructor aided	Approximately 1.5 min	Platform not specified, administered on iPad	United States of America (English)
Itremorsense	Yes	Yes	Unknown	Android OS and iOS; Web-based	Greece
Kassavetis et al	No	Instructor explained each task	Approximately 5 min	Android OS	United Kingdom (English)
Smartphone Tapper (SmT)	Yes	Instructor aided	Approximately 1 min	Android OS	South Korea (English)
STOP (Sentient Tracking of Parkinson’s)	Unknown	Yes	Unknown	Android OS and iOS	Finland and United Kingdom (English)
Composite tools					
Elevatems	Unknown	Yes	Approximately 5 min	iOS	United States of America (English)
FAST-ED App	Unknown	Unknown	Unknown	Unknown	Germany
ICTUS3R	Yes	Yes	Approximately 2 min	Android OS and iOS	Italy (Italian)
M.A.L	Yes	Instructor explains first. Self-assessment after	Approximately 5 min	Android OS	United States of America (English)
MICK App	Unknown	Instructor aided	Unknown	Platform not specified, administered on a tablet	United States of America (English)
PD Dr	Yes	Yes	Approximately 5 min	Android OS	United States of America (English)

The technical properties of the tools are described in Table 2. Of the total, 15 tools (36%) required an internet connection [15, 19, 22, 23, 27, 30, 31, 33, 38, 40, 50, 52, 55, 56, 58], mostly due to real-time data transfer or data upload. At least 26 tools (62%) collect data through self-assessment [15, 17–24, 26, 30–32, 36, 38, 39, 41, 44, 46, 47, 49, 52, 53, 55, 56, 58] without the need for external aid; although some of these highlight the presence of an instructor, mostly at the beginning to explain the procedure. None except one [43] of the included tools required further expertise of a clinician to interpret the output of the data collected. A total of 26 tools (62%) were available in English [15–22, 25, 27–31, 36, 37, 39–44, 53, 56–58], with only 4 (10%) of the tools being available in more than one language [20, 29, 31, 44]. Only 18 tools (43%) were validated in a given population [17, 18, 25, 26, 29–32, 37, 38, 40, 42–44, 51, 52, 54, 56] and another 10 (24%) partially validated against similar measures [15, 16, 20, 21, 23, 36, 39, 41, 45, 58]. Most tools were available on mobile software (e.g., a tablet or smartphone), with at least 5 (12%) being mobile smartphone applications that were compatible with both Android OS and iOS platforms [19, 25, 29, 44, 45]. The remaining tools, which were not available for mobile, were developed for a web-based platform and accessible through a browser.

Information on how to access the eHealth tools was often incomplete in the scientific paper. We reached out to each of the corresponding authors, but only gathered a 24% response rate. Furthermore, of the tools where corresponding authors provided additional information on accessibility, at least one had yet to have a consumer-ready version. Information on tool accessibility can be found in Table 3. By the end of the process, the authors were able to identify Uniform Resource Locators (URLs) for 22 (52%) tools, either to an application store, database, or website [15, 16, 19, 23–25, 27–29, 38, 40–46, 50, 51, 53–55].

**Table 3:** Accessibility data with tool status and available hyperlinks

Tool Name	Source of Funding	Licensing	Last Maintained*	Maintenance Date Info	Availability	URL
ElevateMS	Private	Non-Profit Organisation	Unknown	Unknown	Active Website	Website
iTUG	Private	Proprietary	Mar 3, 2019	App Store Update	Active – New Name	App Store
Bourke et al	Private	Unknown	Unknown	Unknown	Unknown	Unknown
HOKOUKEISOKU-APP	Private	Unknown	Unknown	Unknown	Unknown	Unknown
Itremorsense	Public	Open Source	Aug 25, 2015	APK Date of Publishing	Active APK and Github	APK Github
Smartphone Tapper (SmT)	Public	Open Source	Jun 28, 2016	Github	Active Github	Github
Karolinska WakeApp (KWA)	Public	Open Source	Mar 31, 2021	Github Update	No consumer-ready version available; Active Github	Github
DementiaTest	Public	Proprietary	Dec 1, 2022/Dec 10, 2022	App Store Update/Play Store Update	Active	App Store Play Store
MindLAMP	Public	Proprietary	Jul 13, 2022/Oct 19, 2022	App Store Update/Play Store Update	Active	App Store Play Store
STOP (Sentient Tracking of Parkinsons)	Public	Proprietary	Aug 30, 2019	App Store Update	Active	App Store
EVO Monitor	Public	Proprietary	Unknown	Unknown	Active – Upon Request	NA
Linder et al	Public	Proprietary	Unknown	Unknown	Developer Website Accessible	Developer Website
iVitality	Public	Unknown	Nov 21, 2014	APK Date of Publishing	Active APK	APK
StrokeVision	Public	Unknown	Jun 2, 2017	APK Date of Publishing	Active APK	APK
Digital TMT Black & White (dTMT-B&W)	Public	Unknown	Unknown	Unknown	Unknown	Unknown
SMART	Public	Unknown	Unknown	Unknown	Unknown	Unknown
TabletWebApp	Public	Unknown	Unknown	Unknown	Unknown	Unknown
UX-TMT	Public	Unknown	Unknown	Unknown	Unknown	Unknown
M.A.L	Public	Unknown	Unknown	Unknown	Unknown	Unknown
MICK App	Public	Unknown	Unknown	Unknown	Unknown	Unknown
Adaptive Cognitive Evaluation (ACE)	Public & Private	Proprietary	Apr 21, 2022	App Store Update	Active	App Store
NIHTB-CB	Public & Private	Proprietary	Jul 11, 2022	App Store Update	Active	App Store
Oxford Cognitive Screen-Plus (OCS-Plus)	Public & Private	Proprietary	May 3, 2022	App Store Update/Play Store Update	Active	Website App Store Play Store
6WT app	Public & Private	Proprietary	Mar 19, 2020/Sep 23, 2019	App Store Update/Play Store Update	Active	App Store Play Store
ICTUS3R	Public & Private	Proprietary	May 24, 2022	Play Store Update	Active	Website Play Store
Babyscreen	Public & Private	Proprietary	Unknown	Unknown	Active – Upon Request	Website
C3-PAD	Public & Private	Unknown	Unknown	Unknown	Unknown	Unknown
FallSkip	Public & Private	Unknown	Unknown	Unknown	Unknown	Unknown
MOST-96120	Unknown	Proprietary	Mar 28, 2018	App Store Update	Active	Website
APP-Coo-Balance-Test	Unknown	Proprietary	Sep 13, 2022	Play Store Update	Active	App Store Play Store
SagittalMeter Pro	Unknown	Proprietary	Aug 26, 2019	Play Store Update	Active	Play Store
FAST-ED App	Unknown	Proprietary	Nov 10, 2022	App Store Update	Active – New Name	App Store
Encephalog	Unknown	Proprietary	Dec 7, 2022	App Store Update	Active – Successor App	App Store
Su et al	Unknown	Unknown	Unknown	Unknown	Unknown	Unknown
Voxtester	Unknown	Unknown	Unknown	Unknown	Material Available Upon Request	NA
Cognition Reaction (CoRe)	Unknown	Unknown	Unknown	Unknown	Unknown	Unknown
FatigueApp	Unknown	Unknown	Unknown	Unknown	Unknown	Unknown
Mobile Cognitive Screening	Unknown	Unknown	Unknown	Unknown	Unknown	Unknown
FiMS	Unknown	Unknown	Unknown	Unknown	Unknown	Unknown
Kassavetis et al	Unknown	Unknown	Unknown	Unknown	Unknown	Unknown
PD Dr	Unknown	Unknown	Unknown	Unknown	Unknown	Unknown

^*^Last checked: April 6th, 2023

Out of a total of 16 studies solely publicly funded, at least 10 tools were still accessible at the time of the review [19, 21, 23–25, 38, 40–43]. Out of the 4 privately funded studies, at least 2 tools were still accessible [50, 53]. Of the 8 studies that received both public and private funding, at least six tools were still accessible [15, 16, 28, 29, 44, 55]. The remaining studies disclosed no external funding or had no source of funding information available, with at least 6 tools still accessible [27, 33, 45, 46, 51, 54]. All of the 17 tools that were found to have proprietary licensing (i.e., owned by a private entity or corporation) were still accessible at the time of this review [15, 16, 19, 21, 25, 27–29, 41, 42, 44–46, 50, 51, 54, 55]. One tool had a license belonging to a Non-Profit Organisation [53], and 3 tools were open source [24, 38, 40], all still accessible at the time of data collection.

## Interpretation

This systematic review mapped a total of 42 eHealth software tools that assess one or more neurological signs and/or symptoms, potentially useful for research purposes. The most targeted neurological domain was cognitive function, followed by tools to assess gait, balance and coordination. Interestingly, 6 tools that assess a combination of symptoms and signs were also identified: these were designed to monitor the neurological function in patients affected by specific conditions, i.e., Parkinson’s disease [56, 58], Multiple Sclerosis [53], stroke [54, 55], or consequence of concussion [57]. Relatively less frequent were the tools assessing motor function alone, or cranial nerves.

The disproportionally higher number of tools assessing cognitive function might be due to the fact that cognitive impairment is a frequent manifestation of several late-stage neurological conditions [59, 60]. In addition, it may be easier to transpose a pen-and-paper test to a digital format, in some cases even improving performance in data collection compared to their analogue counterparts [61]. Some neurological domains, such as cranial nerve functions (e.g., facial symmetry, swallowing…), and sensation (e.g., pain, deep sensation), appear seemingly underrepresented in the reviewed studies. This is an important gap for population-based research, where peripheral neuropathies associated with metabolic syndrome [62], and pre-clinical stages of diabetes [63], in particular in the obese population, might go under detected. A tool aimed at screening neurological symptoms for research purposes in the general population would ideally also cover these domains.

While some tools have been either fully or partially validated, facilitating implementation in real-world contexts, the heterogeneity of the description and reporting of the included tools was very high. Some items were described, but testing in a population was not reported, limiting their potential applicability. Other studies reported tools used in clinical settings with patients, as opposed to the general population; however, these were included in this systematic review as they were deemed useful for epidemiological research. In addition, while approximately half of the described tools were available in English, only a very small proportion was available in more than one language, adding to the challenge of performing epidemiological research beyond English-speaking populations.

A notable finding in this systematic review was the scarcity of tools specifically designed for children. Only one tool targeted a young paediatric population [16]. This could partly be attributed to ethical considerations and boundaries that make research on children more complex and challenging [64]. Nevertheless, these hurdles should not deter researchers from focusing on developing age-appropriate tools for children. There is a pressing need to bridge this gap in the field and develop more child-focused tools, designed considering ethical and developmental aspects, to better serve this population group in research settings. No study specifically assessed the ability of the elderly to use eHealth, despite some articles reporting a mean age over 65 for their samples [26, 30, 31, 36, 41, 58]. Previous studies show that this age group experiences higher difficulty working with digital tools [65, 66]. Alongside the expansion of eHealth, a greater emphasis on digital literacy is often promoted, especially since it has been highlighted that eHealth literacy programs have been well received by the elderly in general, both in the form of multimedia training and as paper-based training [67].

Only one tool presented an attempt at cross-cultural validation [29] e.g., the acceptability, feasibility and correct interpretation of outcomes in populations with different cultural norms, including beliefs towards disease, different levels of literacy, or trust in technology, by validating the tool in the different cultural contexts of Central Europe and South Africa [29, 68]. Cross-cultural validation is particularly relevant considering that in some cultures the origin of neurological signs and symptoms in particular, such as seizures or tremor, is often attributed to supernatural causes or prejudiced views (i.e., demonisation and witchcraft) [69, 70]. With the increasing availability of smartphones, eHealth tools could enable data collection for epidemiological research in previous hard-to-reach environments or populations. However, this will not be problem free and additional strategies such as for example involving relevant stakeholders such as policymakers, will be likely needed, as some behavioural and technological barriers still persist in many populations [10, 71–73].

During the review process, the authors searched online for the tools, their original authors, and developers. Access was often a challenge due to missing URLs in papers, missing information on whether the tool was still active or discontinued, and the fact that some of the tools did not have a specific name, had been since renamed or had a successor app that was named differently or looked visually different. These findings replicated previous systematic reviews experiences on app-based research, in the broader healthcare sector. For example, Montano and collaborators [74] reviewed 26 papers on mobile triage applications, of which only 13 (50%) could be identified on the basis of the paper, and only two were still accessible via Google Play Store at the time the review was conducted. In addition to the lack of information to find the tools, the unresponsiveness of the authors posed yet another challenge to accessibility. The inaccessibility of many research applications shortly after the related paper is published is especially relevant in light of the so-called *replication crisis* [75], in this case highlighting the need for accountability and transparency beyond the peer-review process.

The heterogeneity in study design captured by this systematic review suggests that often researchers did not publish the description of the tool they have devised together with its validation as a separate paper (see for example [76] and [77]), but already in the context of the study they are conducting. This inevitably reduces the room for the description of the technical property of the eHealth tools (e.g. its technical design, functionality, implementation, and maintainability) in these papers. When this happens, the specific application is considered as a sufficient method, rather than a required one, meaning that the chosen tool can fulfil the research objective, but can be replaced with another similar application. This reduces considerably the consistency across studies and the ability of pooling or meta-analysing results. Interestingly, the comparison of functionally similar but independently developed software products with small but important differences in design or engineering may introduce errors distorting data collection and biasing data comparison [78]. In general, variations in technology components that are implemented together, or variations in the strategy for their implementation reduce replication fidelity [78]. Most of the tools captured in this systematic review were created in the function of a broader research project, or in preparation for it. The development process was not a primary research objective or method. Separating the app development process from the research question and eliminating any questions related to software engineering from the discussion, compromises replicability, accessibility, and longevity. Unfortunately, it is a common misconception that accessibility and maintenance issues are considered solely as a matter of software engineering. When eHealth tools are specifically developed for a study and their use is a crucial part of the study design, providing information on accessibility and maintenance should not be disregarded as a mere software engineering issue. They must be thoroughly planned and addressed to ensure the replicability of the findings. In this mapping exercise, studies which had a combined source of public and private funding were those most likely to maintain their eHealth tool, in terms of availability and accessibility until the time of this review. However, licensing models were found to be essential for longevity: when the authors and developers of the tool incorporate a strategy of private ownership, either via a company or person, the tools are more likely to remain active. This was evidenced by the fact that all 16 tools that reported private ownership as licensing were still active and accessible at the time of data extraction.

It was not possible to assess the costs of the eHealth tools as such, or in function of their longevity, given a lack of relevant information. Longevity of tools depends mainly on their maintenance strategy to make them compatible with the fast developing and updating mobile technology. Implementing an adequate and lasting maintenance strategy is key to increasing the longevity of eHealth. Challenges of implementing eHealth in real-life contexts, such as the need for it to be more interactive and interoperable, designed to be able to fit multiple contexts, consumers, and providers [79] are well known. However, the ability of eHealth tools to be preserved and usable after development is often overlooked by the scientific literature. By disregarding proper maintenance strategies of eHealth tools, authors may indirectly be raising further challenges to the advancement of eHealth research, development and implementation, at least in the long term. We foresee two main possible strategies that could match costs with longevity. One possible option would be that upcoming eHealth takes into account accessibility and shareability (i.e., making their code open source) so that the scientific and developer community may contribute to keeping eHealth active and usable. Conversely, in the case of proprietary tools, having a designated team that regularly updates the tool and focuses on platform stability appears to be crucial to preserving it over time. However, assuring a maintenance strategy may require constant acquisition and allocation of funds. It is important that the implementation of strategies to promote longevity are established and clarified since the very beginning of the development of eHeatlth tools (i.e., the design phase), to ensure a feasible plan for longevity. Furthermore, future research should focus on producing a standardised measure to assess eHealth, similar to the existing Mobile App Rating Scale (MARS) [80], with the ability to address tool longevity (i.e., accessibility, shareability, costs, ownership, maintenance strategy, etc.).

Given the high number of papers retrieved matching inclusion and exclusion criteria, this review only included software tools. Software with incorporated Artificial Intelligence has been excluded to avoid capturing tools aimed at categorising disease severity or aiding a formal clinical diagnosis. Maintaining the focus on research allowed to map tools to be potentially used for data collection in the field, screening for neurological impairment.

It is important to note that some of these studies and tools focused on collecting signs and symptoms (e.g., tremor) referred to one neurological disease in particular (e.g., Parkinson’s disease). This implies that only symptoms frequently reported by patients with that specific condition are assessed. However, this may not limit the ability of the tool to assess the same set of symptoms in patients with other conditions and in different settings, or in the general population, as pointed out by some of the authors [21, 40]. Nonetheless, the lack of validation of the captured tools is still an ongoing challenge within the eHealth field, representing one of the main barriers to their use. The vast number of studies focusing on proposing and/or developing such tools is not matched by an equivalent number of reports of their validation and application in real-life contexts, with very few being fully validated. Furthermore, the heterogeneity of validation and methods to measure reliability makes it more difficult to draw comparisons. The use of gold standards, combined with appropriate comparison groups (i.e. healthy vs. impaired population), could be a potential solution to reduce heterogeneity of validations. &

## Conclusions

eHealth represents a unique opportunity for researchers, to collect data in the field at contained costs. However, eHealth development appears to often neglect the needs of the population it targets, leading to higher heterogeneity, and lesser validity and reliability. It also appears to disregard the implementation of strategies to keep the tools active over time. Establishing rigorous standards to guide the development of eHealth is increasingly vital in guaranteeing its success. This study mapped existing eHealth software tools aimed at assessing neurological signs and symptoms in populations outside the clinical setting. The mapping and tool descriptions can be used as a guide for neuroepidemiological research. This mapping exercise highlighted the high heterogeneity and low comparability of existing tools, which hamper their use for a much needed, new unique eHealth software, able to screen a wider range of signs and symptoms in population-based studies, for research purposes. This review also emphasises the need to produce more replicable and accessible eHealth research.

## Data Availability

All data is available within the article and supplementary material.
