# Correction to: eHealth tools to assess the neurological function for research, in absence of the neurologist – a systematic review, part I (software)

**DOI:** 10.1007/s00415-024-12558-z

**Published:** 2024-09-17

**Authors:** Vasco Ribeiro Ferreira, Esther Metting, Joshua Schauble, Hamed Seddighi, Lise Beumeler, Valentina Gallo

**Affiliations:** 1 Department of Sustainable Health, University of Groningen, Campus Fryslân, Wirdumerdijk 34, 8911 CE Leeuwarden, The Netherlands; 2 Faculty of Economics and Business, University of Groningen, Groningen, The Netherlands; 3 University Medical College Groningen, Groningen, The Netherlands; 4 Department of Knowledge Infrastructure, University of Groningen, Campus Fryslân, Leeuwarden, The Netherlands; 5 Department of Psychology, Faculty of Behavioural and Social Sciences, University of Groningen, Groningen, The Netherlands; 6 Department of Intensive Care, Medical Centre Leeuwarden, Leeuwarden, The Netherlands

**Correction to: Journal of Neurology (2024) 271:211–230** 10.1007/s00415-023-12012-6

In the original version of this article, in table 3 hyperlinks in the tool description are missing.

The original article has been corrected.

